# Identification of small molecules that disrupt vacuolar function in the pathogen *Candida albicans*

**DOI:** 10.1371/journal.pone.0171145

**Published:** 2017-02-02

**Authors:** Helene Tournu, Jennifer Carroll, Brian Latimer, Ana-Maria Dragoi, Samantha Dykes, James Cardelli, Tracy L. Peters, Karen E. Eberle, Glen E. Palmer

**Affiliations:** 1 Department of Clinical Pharmacy, Division of Clinical and Experimental Therapeutics, College of Pharmacy, University of Tennessee Health Sciences Center, Memphis, Tennessee, United States of America; 2 Department of Medicine, Feist-Weiller Cancer Center, Louisiana State University Health Sciences Center, Shreveport, Louisiana, United States of America; 3 Department of Microbiology, Immunology and Parasitology, School of Medicine, Louisiana State University Health Sciences Center, New Orleans, Louisiana, United States of America; New Mexico Veterans Healthcare System, UNITED STATES

## Abstract

The fungal vacuole is a large acidified organelle that performs a variety of cellular functions. At least a sub-set of these functions are crucial for pathogenic species of fungi, such as *Candida albicans*, to survive within and invade mammalian tissue as mutants with severe defects in vacuolar biogenesis are avirulent. We therefore sought to identify chemical probes that disrupt the normal function and/or integrity of the fungal vacuole to provide tools for the functional analysis of this organelle as well as potential experimental therapeutics. A convenient indicator of vacuolar integrity based upon the intracellular accumulation of an endogenously produced pigment was adapted to identify **V**acuole **D**isrupting chemical **A**gents (**VDA**s). Several chemical libraries were screened and a set of 29 compounds demonstrated to reproducibly cause loss of pigmentation, including 9 azole antifungals, a statin and 3 NSAIDs. Quantitative analysis of vacuolar morphology revealed that (excluding the azoles) a sub-set of 14 VDAs significantly alter vacuolar number, size and/or shape. Many *C*. *albicans* mutants with impaired vacuolar function are deficient in the formation of hyphal elements, a process essential for its pathogenicity. Accordingly, all 14 VDAs negatively impact *C*. *albicans* hyphal morphogenesis. Fungal selectivity was observed for approximately half of the VDA compounds identified, since they did not alter the morphology of the equivalent mammalian organelle, the lysosome. Collectively, these compounds comprise of a new collection of chemical probes that directly or indirectly perturb normal vacuolar function in *C*. *albicans*.

## Introduction

Recent decades have seen a dramatic increase in invasive fungal infections, including life-threatening disseminated as well as debilitating mucosal diseases [1–3]. Current antifungal therapies act upon a limited number of targets within the fungal cell, and have serious limitations including patient toxicity, limited spectrum of activity, restrictive formulations, and the emergence of resistant fungal isolates [4–9]. Consequently, mortality rates associated with disseminated fungal infections remain disturbingly high [2,10].

Several recent studies with the human pathogens *Candida albicans*, and *Cryptococccus neoformans*, have established the vital role played by the fungal vacuole in supporting infection of the mammalian host. Mutants of either fungus with significant vacuolar defects are unable to cause lethality in mouse models of disseminated disease [11–14], and in some cases unable to survive within the mouse tissues. Normal vacuolar function has also been implicated in the ability of *Candida glabrata* and *Histoplasma capsulatum* to cause disease [15,16]. The essential role played by the vacuole during host colonization and invasion in these divergent pathogens, underscores the central importance of this organelle to fungal pathogenesis. Defects in vacuolar function result in fungal susceptibility to host derived stresses as well as the loss of virulence related attributes that are associated with host damage. In *C*. *neoformans*, disruption of normal vacuolar function diminishes melanization, urease activity, polysaccharide capsule formation and the ability to grow at 37°C [17], each of which significantly impact pathogenicity. On the other hand, vacuole deficient *C*. *albicans* mutants have profound defects in invasive hyphal growth [11,13,18], which is also crucial for pathogenicity [19, 20]. Thus chemical agents that perturb vacuolar function, should impair fungal pathogenesis on multiple levels—significantly undermining fungal viability *in vivo*, as well as the expression of specific traits required for host damage and/or disease progression.

The purpose of this study was to identify chemical agents that disrupt the normal function and/or integrity of *C*. *albicans* vacuole. We also examined their impact on the integrity of the equivalent organelle in mammalian cells, the lysosome.

## Materials and methods

### Strains and growth conditions

*C*. *albicans* was routinely grown on yeast extract-peptone-dextrose (YPD) agar plates at 30°C, supplemented with 50 μg ml^−1^ uridine when necessary. Selection of *C*. *albicans* transformants was carried out on minimal YNB medium (6.75 g L^−1^ yeast nitrogen base without amino acids, 2% dextrose, 2% Bacto agar) supplemented with the appropriate auxotrophic requirements or with 50 μg ml^−1^ uridine. Screening and secondary assays were conducted in YNB medium supplemented with 5 μg ml^-1^ adenine (unless otherwise stated) at 30°C. Hyphal growth was induced in synthetic complete medium supplemented with 0.2% glucose at 37°C.

### Plasmids

Plasmid pLUX [21] was kindly provided by William Fonzi (Georgetown University), pRSARG4ΔSpeI, pGEMHIS1 and pDDB57 [22, 23], were kindly provided by Dr. Aaron Mitchell (Carnegie Mellon University). Plasmids pKE1 [24] and pKE1-GFP-YPT72 [25] were described previously. Plasmids pKE1-NLUC and pKE1-CPP-NLUC were constructed using a *C*. *albicans* codon-adapted version of the Nano luciferase (Nluc) [26] coding sequence as described in [27]. To facilitate the expression of cytoplasmic mCherry in *C*. *albicans*, the mCherry coding sequence was amplified from plasmid pMG2254 [28], using primer pair mChORFF-EagI and mChORFR-MluI (all oligonucleotides used in this study are listed in S1 Table), and cloned between the EagI and MluI sites of pKE1 to produce plasmid pKE1-mCh.

Plasmid pKE2-GFP-YPT72 was constructed by amplifying the *ACT1pr-GFP-YPT72-ADH1 3’UTR* expression cassette from pKE1-GFP-YPT72 with primers ACT1prF-BamHI and ADH1-3’UTRR-ApaI, and cloning the product between the ApaI and BamHI sites of pGEMHIS1.

### *C*. *albicans* strains and strain construction

All strains used in this study are presented in S2 Table. SC5314 has been described previously [29], CAI4 and CAI8 [30] were kindly provided by William Fonzi (Georgetown University). *C*. *albicans* gene deletion strains were constructed by the PCR-based approach described by Wilson *et al*. [23] and was transformed with DNA constructs by using the lithium acetate procedure [31]. The *vps11Δ/Δade2*^*-/-*^ and *ypt72Δ/Δade2*^*-/-*^ double deletion strains were constructed as follows. Gene deletion cassettes were amplified with primer set YPT72DISF and YPT72DISR or VPS11DISF and VPS11DISR with pDDB57 (recyclable *URA3-dpl200* selection marker) [22] as template. The *ade2*^*-/-*^ strain, CAI8, was then transformed with either the *ypt72Δ*:*URA3-dpl200* or *vps11Δ*:*URA3-dpl200* gene deletion cassettes. Correct integration of either gene deletion cassette was confirmed by diagnostic PCR, using primers URA3INTF2 and YPT72AMPR2 or VPS11AMPR2. The regeneration of *ura3*^*-*^ recombinants was then selected on YNB media supplemented with uridine and 1 μg ml^-1^ 5-FOA [32], before a second round of gene deletion was performed using the same *ypt72Δ*:*URA3-dpl200* or *vps11Δ*:*URA3-dpl200* cassettes. The absence of either the *YPT72* or *VPS11* open reading frame following deletion of both alleles was confirmed by PCR, using primer pair YPT72DETF and YPT72DETR or VPS11DETF3 and VPS11DETR3 respectively. Following a further round of 5-FOA selection, *ura3*^*-*^ recombinants of the double deletion strains and the CAI8 parental strain were each transformed with NheI linearized pLUX to restore uracil prototrophy. Correct integration of pLUX fully reconstitutes the *URA3-IRO1* loci, circumventing the well-described positional effects of *URA3* integration [33]. This was confirmed by the presence of a 2.2 Kb product following PCR with primers LUXINTDETF/R. This yielded congenic *ade2*^*-/-*^, *ypt72Δ/Δade2*^*-/-*^and *vps11Δ/Δade2*^*-/-*^strains of *C*. *albicans*.

The *GFP-YPT72* fusion expressing strain was obtained by transforming CAI4 with NheI linearized pKE1-GFP-YPT72 digested with NheI. For the Cellomics based analysis of vacuolar size and shape the strain GP100 was used, which expresses both GFP-Ypt72p and cytoplasmic mCherry. GP100 was made by sequentially transforming BWP17 with NheI cut pKE1-mCh (*URA3* selection), NruI cut pKE2-GFP-YPT72 (*HIS1* selection), and ClaI cut pRSARG4ΔSpeI to produce a prototrophic dual tagged strain.

### Compound collections

The following libraries of drug or drug-like small molecules were screened for VDA activity: 1) Prestwick Chemical Library (1120 compounds), comprises of a collection of active small molecules selected for high chemical and pharmacological diversity as well as for known bioavailability and safety in humans. 2) Prestwick Phytochemical Library (320 compounds), comprises of natural products mostly derived from plants. This library represents a chemically diverse selection of flavonoids, alkaloids, terpenes and coumarins. 3) GreenPharma Natural Products Library (480 compounds), comprises of a set of chemically diverse, natural and drug-like molecules selected from plants, fungi and micro-organisms.

### Pigmentation assay and compound screening

From 1 mM DMSO stocks, each library chemical was diluted to 10 μM in YNB broth + 5 μg/ml adenine, and 100 μl seeded to the wells of a round bottomed 96-well plate. Strain CAI8LUX1 (*ade2*^*-/-*^) was grown overnight in YPD broth, diluted 1:200 in YNB broth + 5 μg/ml adenine, and 100 μl of the cell suspension added to each well of the 96-well plate. This gave a final compound concentration of 5 μM, with 0.5% DMSO. Control wells (minus drug) had an equivalent concentration of DMSO alone. Assay plates were then incubated in a humidified incubator at 30°C for 48 hours, plates briefly spun in a benchtop centrifuge at 750 rpm to collect the cells at the bottom of the plate before pigmentation was quantified using a Biotek Synergy imaging plate reader with excitation at 488 nm and emission detected at 569 nm, and growth measured using OD_600nm_. For each well, the pigment fluorescence was normalized for growth by dividing by the corresponding OD_600_ value. The mean and standard deviation of the fluorescence were calculated from the minus drug wells for each plate (n = 8), and used to express the normalized fluorescence of each well as a Z-score. Assays were performed in duplicate, and compounds causing significant reduction in normalized fluorescence (Z ≤ -2.5) selected for further analysis.

### CMAC accumulation measurement

The assay of vacuolar accumulation of the cell-tracker blue dye (Invitrogen), CMAC, was optimized for use in a 96-well format. In brief, cells were grown and diluted as described for the pigmentation assay with SC5314 and YNB as the tested strain and medium. For the dose-response assay, serial dilutions of the compound stocks were made in order to keep the final concentration of DMSO at 0.5%. Cells were incubated at 30°C in the presence of compounds or DMSO for 24 h. CMAC was added at a final concentration of 25 μM. After mixing, plates were incubated at room temperature in the dark for 30 min. CMAC accumulation was then measured from cell pellets. Fluorescence was measured at 354 nm excitation and 469 nm emission with a 20 nm bandwidth and growth quantified as OD_600nm_ using a Biotek Synergy imaging plate reader.

### Luciferase assay

The luciferase release assays using *C*. *albicans* strains expressing either Nano luciferase (Nluc—cytoplasmic) or Cpy1^1-129^-Nluc (vacuolar targeted) were performed as previously described [27], with a slight modification to add the compounds. Each compound was added at either 5 or 25 μM (final DMSO concentration 0.5%), or 0.5% DMSO (minus drug control) to the cell suspensions in a 96-well format. After 24 h of incubation at 30°C, plates were centrifuged and fifty microliters of each of the culture supernatant was then transferred to a fresh, flat-bottom white 96-well plate, and the amount of Nluc activity was determined using the Nano-Glo luciferase assay reagent (Promega Corporation) per the manufacturer's instructions. Luminescence in each well was then measured by using a BioTek Synergy imaging plate reader. Growth in each well from the overnight incubation was also determined by measuring the OD600 of each well, and each luminescence reading normalized to the corresponding OD600 reading. An additional control for vacuolar trafficking defects was provided by the vacuolar protein sorting mutant *vps21Δ/Δ* transformed with the same vectors pKE1-NLUC and pKE1-CPP-NLUC.

### Cellomics analysis of the fungal vacuoles

YEPD broth was inoculated with *C*. *albicans* strain GP100 (expressing the *GFP-YPT72* fusion and cytoplasmic mCherry) and grown overnight at 30°C, 180 rpm. Following 1:200 dilution in YNB medium, test compounds were added to a final concentration of either 5 or 25 μM, and 100 μL of each cell suspension seeded to the wells of a U-bottom 96-well plate (Corning #3799). Cultures treated with an equivalent amount of DMSO solvent (0.5%), or 5 μM fluconazole provided negative and positive controls of vacuolar disruption respectively. Following overnight incubation at 30°C plate cultures were resuspended and diluted 1:18 with fresh YNB medium, before 50 μL was transferred to a 96-well glass bottom assay plate (Corning #4580). Assay plates were spun at 400-500g for 5 min at room temperature, then stored in the dark for 1 hour to allow yeast to settle to bottom of the wells. Images were acquired and analyzed on a Thermo Fisher Cellomics Array Scan VTI HCS Reader using HCS Studio^™^ Cell Analysis Software. The filter settings for each dye were BGRFR 549–15 for yeast cytoplasm (mCherry-Channel 1) and BGRFR 485–20 for yeast vacuole (GFP-Channel 2). Images were acquired using a 40X objective for a total of 16 different fields in each well. The Spot Detector BioApplication was used to identify the cells in Channel 1 and to identify and measure the yeast vacuole size and number in Channel 2.

### Mammalian cell lines

Human immortalized keratinocytes HaCaT were a gift from Dr. Rona Scott (LSUHSC-Shreveport). Cells were maintained in Glutamax DMEM supplemented with 10% fetal bovine serum and subcultured upon reaching 80% confluence.

### Reagents and antibodies

Reagents and antibodies for immunofluorescence and staining of mammalian cell lines were purchased as follow: LAMP1 antibody (H4A3-s, Developmental Studies Hybridoma Bank, Iowa State University), Fluorescently conjugated donkey anti-mouse (Jackson Immunoresearch, West Grove, PA), Phalloidin-488, DAPI, and Slow Fade Gold Reagent (Life Technologies, Carlsbad, CA), Tubulin antibody (1:20,000, Neomarkers, Freemont, CA), Cathepsin B antibody (1:500 Santa Cruz Biotechnology, Dallas, TX).

### Immunofluorescence

Cells were fixed with 4% paraformaldehyde in phosphate buffered saline (PBS) for 20 minutes at room temperature. Primary antibody was incubated 1:200 in BSP (0.25% bovine serum albumin, 0.1% saponin, PBS) for one hour at room temperature. Fluorescently conjugated secondary antibody was incubated 1:200 in BSP for one hour at room temperature. Phalloidin 1:200 in BSP for 20 minutes at room temperature was used to visualize the actin cytoskeleton. Cells were then incubated with DAPI for 20 minutes in BSP, prior to mounting sides with Slow Fade Gold Reagent.

### Cellomics analysis of lysosomes

HaCaT cells were plated at 10,000 cells/well and treated with compounds in complete media for 25 hours. Immunofluorescence was performed using DAPI to detect the cell nucleus and anti-LAMP1 antibody to detect lysosomes. Images were acquired and analyzed on a Thermo Fisher Cellomics ArrayScan VTI HCS Reader using HCS Studio^™^ Cell Analysis Software. The filter settings for each dye were BGRFR 386–23 for nucleus (Channel 1) and BGRFR 549–15 for lysosomes (Channel 2). Images were acquired using a 40X objective for a total of 20 different fields in each well. The Spot Detector Bioapplication was used to identify the objects in Channel 1 and to measure the lysosomes size and number in Channel 2.

## Results

### Red pigmentation of *Candida albicans ade2Δ/Δ* mutants can provide an indicator of vacuolar integrity

**Fig 1:**
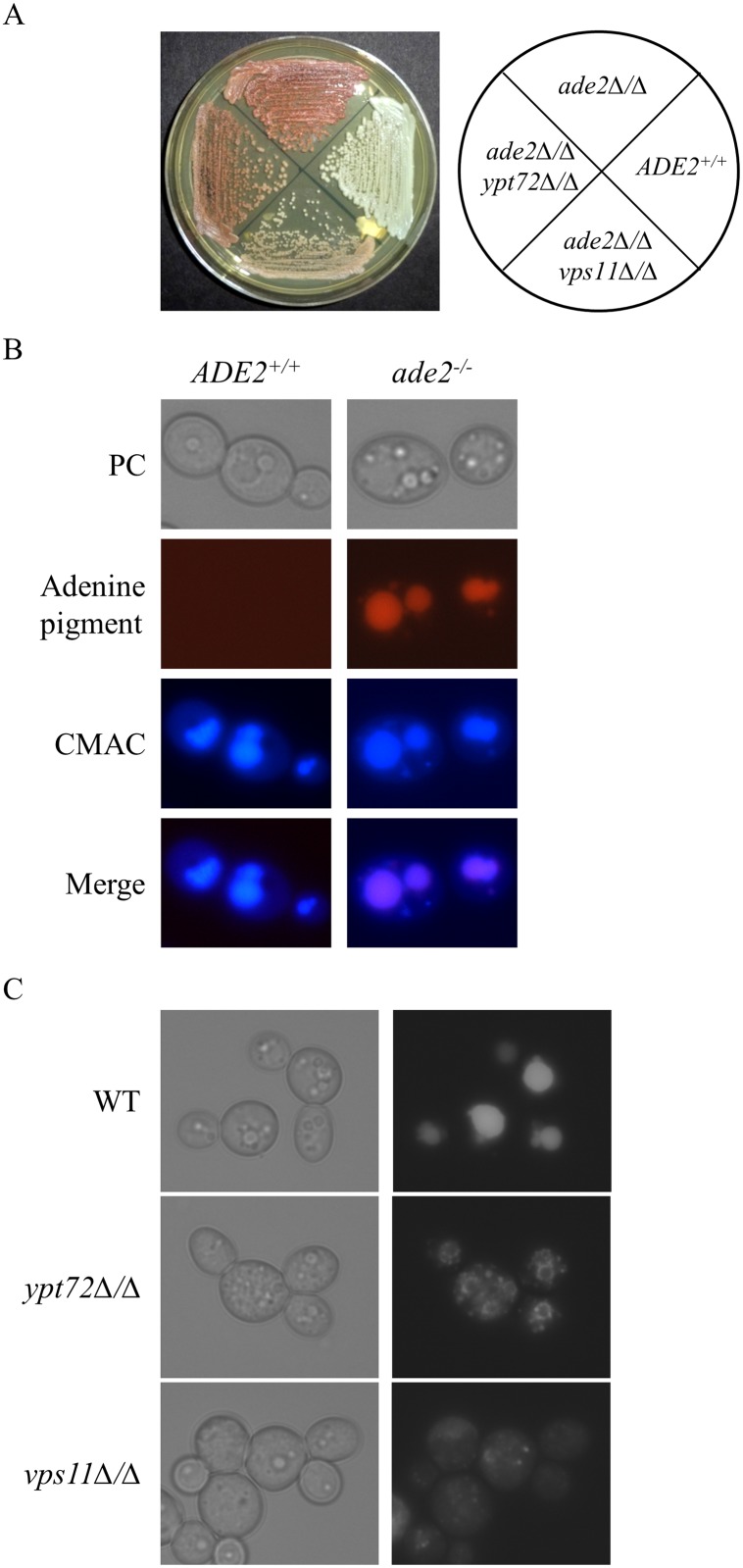
*C. albicans ade2* mutant produces a fluorescent pigment that localizes within the fungal vacuole. (A) *ADE2*^*+/+*^ (SC5314), *ade2*^*-/-*^ (CAI8LUX1), *ade2*^*-/-*^*ypt72Δ/Δ* (C8ypt72Δ/Δ) and *ade2*^*-/-*^*vps11Δ/Δ* (C8vps11Δ/Δ) mutant strains were streaked to YPD agar and incubated for 48 hours prior to imaging. (B) *ADE2*^*+/+*^ and *ade2*^*-*/-^ strains were grown in YNB + 5 μg/ml adenine overnight, stained with CMAC and examined by fluorescent microscopy using TRIT-C and DAPI filter sets. (C) *ade2*^*-/-*^ (WT), *ade2*^*-/-*^*ypt72Δ/Δ* and *ade2*^*-/-*^*vps11Δ/Δ* mutant strains were grown in YNB + 5 μg/ml adenine overnight and cells examined by fluorescent microscopy using TRIT-C filter sets.

Certain *Saccharomyces cerevisiae* mutants blocked in adenine biosynthesis accumulate a pigmented biosynthetic intermediate, 5’-phosphoribosylaminoimidazole (AIR), that gives them a distinctive red coloration when adenine is supplied in limiting quantities [34–37]. The AIR pigment is conjugated to glutathione and pumped into the fungal vacuole where it accumulates, and has been used as an endogenous fluorescent marker to examine vacuolar morphology [37]. Furthermore, yeast mutants with significant defects in vacuolar biogenesis are reported to have reduced pigment accumulation in the *ade2*^*-/-*^ mutant background [38]. We sought to exploit the intracellular accumulation of AIR to devise a screening assay to identify chemicals that perturb the fungal vacuole, in the prevalent human fungal pathogen, *Candida albicans*. To establish the validity of this approach, we examined a *C*. *albicans ade2*^*-/-*^ mutant [30] (Fig 1A) by fluorescent microscopy, to confirm that the adenine biosynthetic intermediate could be used as an endogenous marker of vacuolar integrity. The *ade2*^*-/-*^ mutant accumulates a red fluorophore not present in *ADE2*^*+*^ strains, typically in one or more large sub-cellular compartments (Fig 1B). The vacuolar marker dye CMAC [39], a membrane permeable coumarin derivative colocalized with the endogenous fluorophore, confirming its accumulation within the fungal vacuole.

We next determined if disruption of vacuolar integrity would affect the bioaccumulation of the red pigment in the *C*. *albicans ade2*^*-/-*^ mutant background. For this we constructed *C*. *albicans* mutants with defects in vacuolar biogenesis by deleting either the *YPT72* or *VPS11* genes in the *ade2*^*-/-*^ strain [14,18,40]. Both vacuolar deficient mutants were visibly less pigmented than the *ade2*^*-/-*^ control strain (Fig 1A). In addition, microscopic inspection using the endogenous fluorophore confirmed the extensive vacuolar morphological defects in both *ade2*^*-/-*^*ypt72Δ/Δ* and *ade2*^*-/-*^*vps11Δ/Δ* mutants versus the *ade2*^*-/-*^ control strain (Fig 1C).

### The pigmented *C*. *albicans ade2Δ/Δ* strain can provide the basis of a high-throughput screening assay of vacuolar function

**Fig 2:**
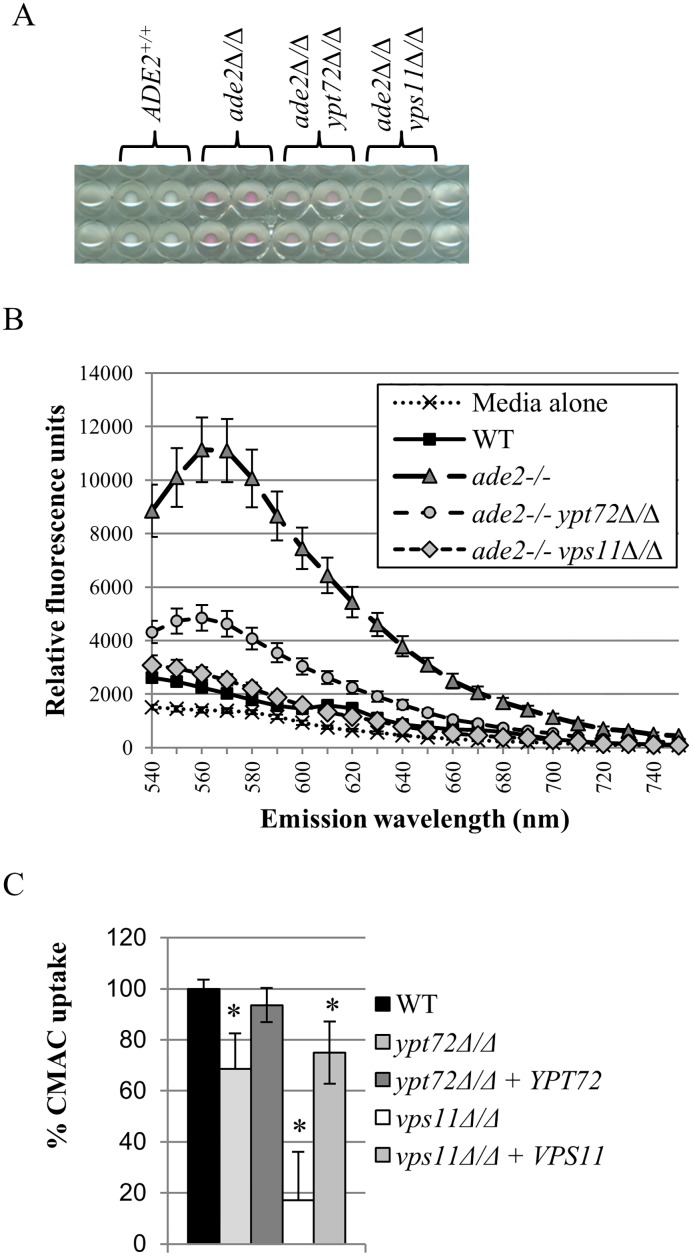
Pigmentation of the *ade2*^*-/-*^ mutant can form the basis of a 96-well plate based screening assay of vacuolar integrity. (A) The indicated strains were suspended at 1 x 10^5^ cells/ml of YNB + 5 μg/ml adenine medium, before 200 μl aliquots were dispensed into the wells of a 96-well plate. The wells were imaged after 48 hrs incubation at 30°C. (B) A spectral scan was performed on the wells of the 96-well plate described in part (A) above, with excitation at 488 ± 10 nm, and fluorescence emission read at the indicated wavelengths. At each wavelength, relative fluorescence is normalized for growth, as measured by OD_600nm_. (C) The indicated strains (all *ADE2*^*+*^) were growth in YNB medium in the wells of a 96-well plate, stained with CMAC, and then CMAC fluorescence detected following excitation at 354 nm and emission at 469 nm. CMAC uptake is normalized for growth, as measured by OD_600nm_, and is the mean of three independent experiments. * p < 0.0001 versus the WT control strain.

We next used a non-pigmented ‘wild-type’ *C*. *albicans* strain (SC5314), an *ade2*^*-/-*^ strain (CAI8LUX1), as well as the *ade2*^*-/-*^*ypt72Δ/Δ* and *ade2*^*-/-*^*vps11Δ/Δ* derivatives, to optimize a 96-well plate based pigmentation assay (Fig 2A). The optimal wavelength for the detection of accumulated pigment was determined using a range of excitation and emission wavelengths (Fig 2B). This revealed that the adenine pigment could be detected over a wide range of excitation/emission wavelengths in the red spectrum, but optimally at excitation 488 ± 10 nm and emission 569 ± 10 nm. Thus these wavelengths were used in subsequent experiments to quantify pigmentation. At these wavelengths both *ade2*^*-/-*^*ypt72Δ/Δ* and *ade2*^*-/-*^*vps11Δ/Δ* cell pellets retained significantly less pigment vs. the *ade2*^*-/-*^ control strain (Fig 2B). When grown under assay conditions in a 96-well plate, comparing the pigmentation of the *ade2*^*-/-*^ strain to that of the *ade2*^*-/-*^*ypt72Δ/Δ* and *ade2*^*-/-*^*vps11Δ/Δ* vacuole deficient strains determined that the pigmentation assay had a Z-prime (Z’) of 0.67 ± 0.05 and 0.67 ± 0.03 (mean ± standard deviation deduced from four independently performed experiments, N = 30 in each experiment) respectively. Since Z’ factors > 0.5 are typically considered acceptable for chemical screening [41], these data suggest the assay is of sufficient quality to provide a feasible chemical screen. Collectively, these studies confirmed that disruption of vacuolar integrity could be detected spectroscopically using the *C*. *albicans ade2*^*-/-*^strain grown in the 96-well format.

### Identification of chemical disruptants of the *C*. *albicans* vacuole

We applied the above assay to screen three libraries of drug, or drug-like small molecules (1920 compounds in total), at a concentration of 5 μM, to identify those that cause loss of pigmentation. In the primary screen, we identified 29 potential **V**acuole **D**isrupting **A**gents (**VDA**’s) that reproducibly inhibited pigment formation (Table 1), an initial hit rate of 1.5%. Interestingly, nine of the hits identified as VDAs belong to the well-characterized azole class of antifungals, which block the synthesis of the membrane lipid ergosterol. We have previously reported that fluconazole disturbs vacuole morphology and function prior to its effect on growth [25]. We therefore focused on the remaining 20 compounds that belong to various classes of drugs, including ionophores, statins and NSAIDs.

**Table 1:** List of VDA compounds.

Hit compound	Library	Compound class
miconazole	Prestwick	azole class
ketoconazole	Prestwick	
clotrimazole	Prestwick	
econazole nitrate	Prestwick	
sulconazole nitrate	Prestwick	
sertaconazole nitrate	Prestwick	
isoconazole	Prestwick	
butoconazole nitrate	Prestwick	
enilconazole	Prestwick	
monensin sodium salt	PhytoChemical	ionophore
3,6-dibromo-purpurogallin	GreenPharma	polyphenol antibiotic
5-(3-furan-2-yl-acryloyl)-2,2-dimethyl-4,6-dioxo-cyclohexanecarboxilic acid methyl ester	GreenPharma	acid methyl ester
vulpinic acid	GreenPharma PhytoChemical	acid methyl ester
ciclopirox ethanolamine	Prestwick	iron chelator salt
fluvastatin sodium salt	Prestwick	statin
methyl benzethonium chloride	Prestwick	quaternary ammonium salt
kinetin riboside	PhytoChemical	ribonucleoside analog
clioquinol	Prestwick	hydroxyquinoline
tolfenamic acid	Prestwick	NSAID
mefenamic acid	Prestwick	NSAID
diflunisal	Prestwick	NSAID
dequalinium dichloride	Prestwick	quaternary ammonium salt
n-methyl-2,4-dihydroxy-3-phenylquinoline	GreenPharma	phenylquinoline
conferone	GreenPharma	sesquiterpene coumarin
ribavirin	Prestwick	ribonucleoside analog
benzbromarone (3-(3,5-dibromo-4-hydroxybenzoyl)-2-ethylbenzofuran)	Prestwick	benzofuran derivative
7,8-dihydroxy-3-(2-methyl-thiazol-4-yl)-chromen-4-one	GreenPharma	benzopyran derivative
halofantrine hydrochloride	Prestwick	phenanthrene class

We first considered that loss of pigmentation could arise not only from impaired accumulation within the vacuole, but also from inhibition of AIR pigment synthesis at a step of the adenine biosynthetic pathway upstream of Ade2p. We therefore applied a secondary measure of vacuolar integrity that is independent of the adenine biosynthetic pathway, specifically the intracellular accumulation of CMAC. A 96-well plate based assay was adapted and validated using the *C*. *albicans ypt72Δ/Δ* and *vps11Δ/Δ* mutants, which accumulate significantly less CMAC than wild-type (Fig 2C). In dose-response experiments (0–50 μM), all except two compounds reduced CMAC accumulation compared to the minus drug controls (S1 Fig), supporting that they disrupt vacuolar function and/or integrity. Ribavirin and kinetin riboside had no effect upon CMAC accumulation. Both are purine nucleoside analogs, and thus may directly or indirectly impinge upon the adenine synthetic pathway to reduce the production of the red pigment. Among the remaining 18 VDA’s, 8 significantly reduced fungal growth (as measured by OD_600nm_) at concentrations that coincided with CMAC reduction, 1 inhibited fungal growth but only at concentrations above that which reduced CMAC accumulation, while 9 had no obvious effect on growth under the conditions the CMAC assay was performed.

### VDA compounds cause a variety of abnormal vacuolar morphologies

**Fig 3:**
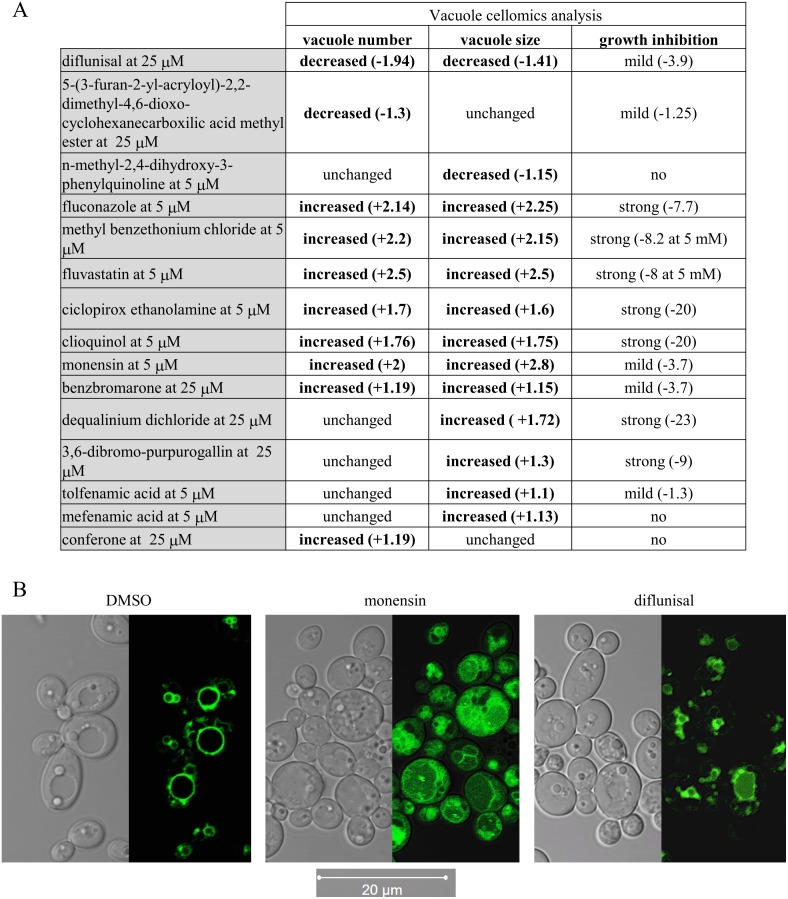
Several VDAs induce abnormal vacuolar morphologies. (A) Bio-imaging cellomics analysis of vacuole number and size and cell count. The *C*. *albicans* strain GP100, expressing cytoplasmic mCherry and vacuolar membrane GFP-*YPT72*, was exposed to each VDA at 5 and 25 μM at 30°C. The numbers of cells and the number and size of the vacuoles were determined for each individual cell present in 16 different fields in each well, and that for 6 wells for each compound. The Spot detector BioApplication (HCS Studio^™^ Cell Analysis Software) was optimized to identify each fungal cell via the mCherry channel, and the number and size of each vacuole, within each cell, via the GFP channel. Numbers in brackets indicate the fold variation relative to DMSO treated cells. Only significant decrease or increase is shown (p-value < 0.0001). (B) Confocal imaging of cells expressing GFP-*YPT72* and treated with 0.5% DMSO, diflunisal at 50 μM, or monensin at 12.5 μM for 24h at 30°C. Images were acquired using a Zeiss LSM 710 laser scanning confocal microscope.

To further investigate the impact of each VDA upon vacuolar integrity, we utilized a *C*. *albicans* strain expressing *GFP-YPT72* fusion and cytoplasmic mCherry (G100 strain; S2 Table). *YPT72* encodes a GTPase of the Rab family, which inserts into the cytoplasmic leaflet of the vacuolar membrane [40], and does not therefore depend upon membrane trafficking for localization. The G100 strain was grown in suspension cultures in presence of 5 or 25 μM of each compound, or with DMSO vehicle alone, and vacuolar morphology analyzed using an automated, high-content imaging platform (Cellomics ArrayScan VTI HCS Reader and HCS Studio^™^ Cell Analysis Software). Fluconazole was used as an internal control for these experiments as we have previously shown that it resulted in severe vacuolar fragmentation [25]. In YNB medium, 14 of the 18 VDAs tested were found to significantly affect vacuole number and/or size (Fig 3A), with a variety of distinct abnormalities observed. These fell into two broad categories, with 3 compounds reducing total vacuolar size and/or area per cell, and 11 compounds increasing total vacuolar area and/or number per cell. Of the 11 VDAs that induced vacuolar fragmentation (increased vacuole number and/or area), 6 were strong growth inhibitors (defined as >50% growth inhibition compared to DMSO control), as was the positive control fluconazole, 3 mildly affected *C*. *albicans* growth and 2 had no effect on growth (Fig 3A). Of the 3 compounds observed to decrease vacuolar size or number (diflunisal, n-methyl-2,4-dihydroxy-3-phenylquinoline and 5-(3-furan-2-yl-acryloyl)-2,2-dimethyl-4,6-dioxo-cyclohexanecarboxilic acid methyl ester), none caused severe growth defects. The NSAID diflunisal was the sole compound to significantly reduce both vacuolar size and number per cell (Fig 3). Thus not all of the observed vacuolar abnormalities coincided with impaired fungal growth. Confocal imaging further illustrates the altered vacuolar organization caused by diflunisal and monensin, as examples of VDAs that caused vacuolar shrinkage and fragmentation respectively (Fig 3B).

### VDAs do not cause vacuolar trafficking defects

Defects in membrane trafficking between the Golgi and the vacuole interfere with the biogenesis of this organelle and can lead to morphological abnormalities [42]. We therefore examined the effect of the 14 VDAs that altered vacuolar morphology, upon Golgi-vacuole trafficking, using an in-house nano luciferase^™^ (Nluc—Promega Corporation) based reporter assay [26,27]. The assay uses a *C*. *albicans* strain in which Nluc is targeted to the fungal vacuole using the pre-pro-peptide of vacuolar carboxypeptidase Y (CPP-Nluc). Perturbation of Golgi-vacuole trafficking leads to missorting of a portion of CPP-Nluc into the secretory pathway, and thus CPP-Nluc activity can be detected extracellularly within the culture supernatant [43,44]. This was confirmed by introducing the CPP-Nluc expression construct into a *C*. *albicans vps21Δ/Δ* mutant [40], blocked in Golgi to vacuole trafficking at the PVC. The mutant secreted typically 30-fold more CPP-Nluc activity that the wild-type control (S2 Fig).

The wild-type CPP-Nluc expressing strain was treated with each VDA, and CPP-Nluc activity in the culture supernatant assayed after 24 hours. To provide an indicator of cell lysis, we performed the same experiment with a second strain expressing a cytoplasmic form of Nluc. CPP-Nluc activity released into the culture supernatant was then normalized to cytoplasmic Nluc activity released into the culture supernatant. As an additional control, we used the fungicidal antifungal amphotericin B (AmB), which causes severe plasma membrane leakage via the formation of ergosterol-dependent aqueous pores in phospholipid bilayers [45]. As anticipated, AmB caused significant release of cytoplasmic Nluc, but not of the vacuolar CPP-Nluc (S2 Fig). None of the VDAs significantly induced the preferential release of CPP-Nluc over cytoplasmic Nluc (data not shown), suggesting they do not specifically target Golgi-vacuole trafficking. Fluvastatin was the only compound tested that caused the significant release of cytoplasmic Nluc after 24 hours (S2 Fig), indicating cell lysis.

### VDAs impact *C*. *albicans* hyphal growth

**Fig 4:**
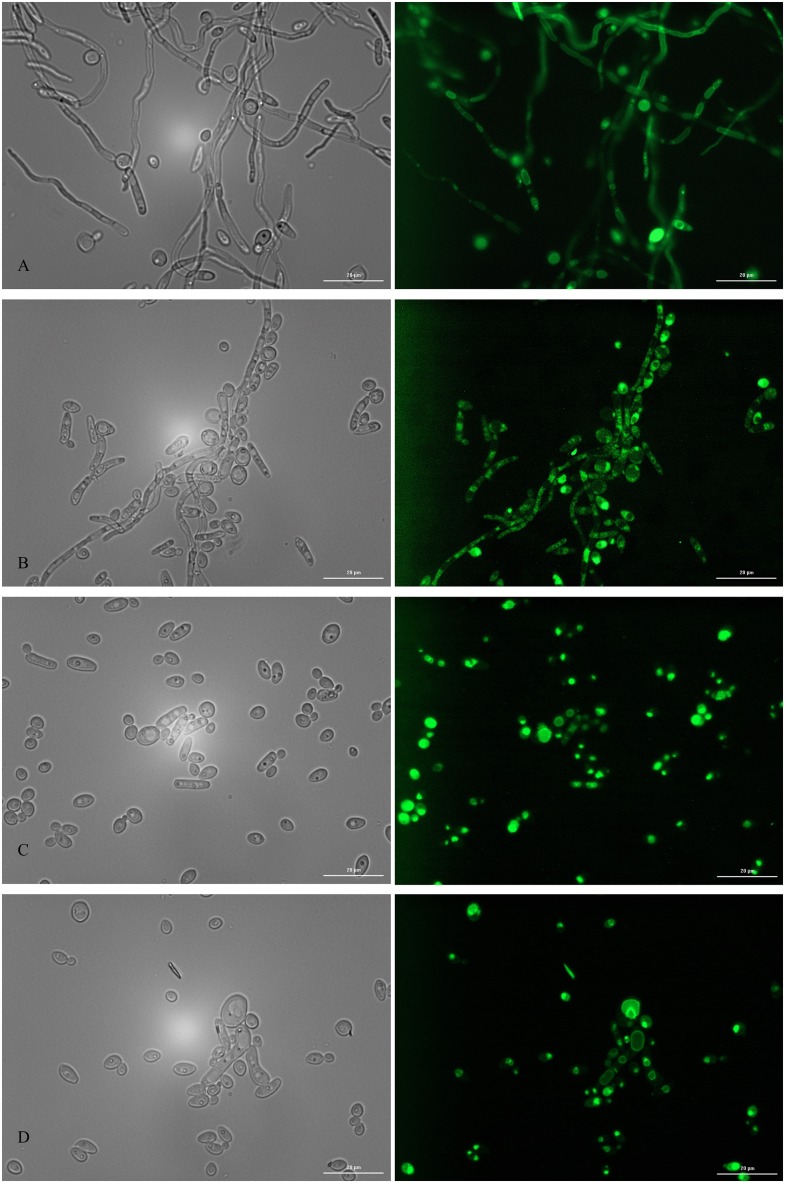
Hyphal growth assay in presence of the 3 NSAIDs in the VDA collection. Hyphal growth was imaged after 24h of growth at 37°C in low glucose conditions using a strain expressing GFP-YPT72. Cells were challenged with 0.5% DMSO (A), 50 μM diflunisal (B), 50 μM tolfenamic acid (C), and 50 μM mefenamic acid (D). Images were acquired using a BioTek Synergy imaging reader at a 60X magnification for bright field imaging (left panels) and fluorescence (eGFP excitation filter of 485/20; right panels). The error bars represent 20 microns.

Defects in vacuolar biogenesis can severely impair *C*. *albicans* capacity to form true hyphae, a growth form which is intimately associated with its pathogenicity [19, 46]. In addition, highly vacuolated cell compartments can often be observed in sub-apical regions of *C*. *albicans* hyphae [47, 48]. We therefore examined the effect of the 14 VDAs affecting vacuolar morphology upon *C*. *albicans* hyphal growth in a glucose-limited complete medium at 37°C. This enabled us to keep a similar pH across all experiments described in this study and avoid the variable effects of serum binding/sequestration on each VDA that would be expected to occur using blood serum to induce hyphal growth. We used the GFP-*YPT72* expressing strain to facilitate analysis of vacuolar morphology in the hyphal cells. In the absence of VDAs (DMSO control), *C*. *albicans* formed elongated and highly polarized hyphal elements with a large proportion of sub-apical cells occupied by vacuoles, as previously described (Fig 4). All 14 VDAs had a negative impact on hyphal formation (S3 Fig), inducing a variety of abnormal morphological forms. Notably the NSAIDs, tolfenamic and mefenamic acids, which cause fragmentation of vacuoles in the yeast form, completely abolished hyphal development (Fig 4).

### Some VDA compounds disturb lysosomal morphology

**Fig 5:**
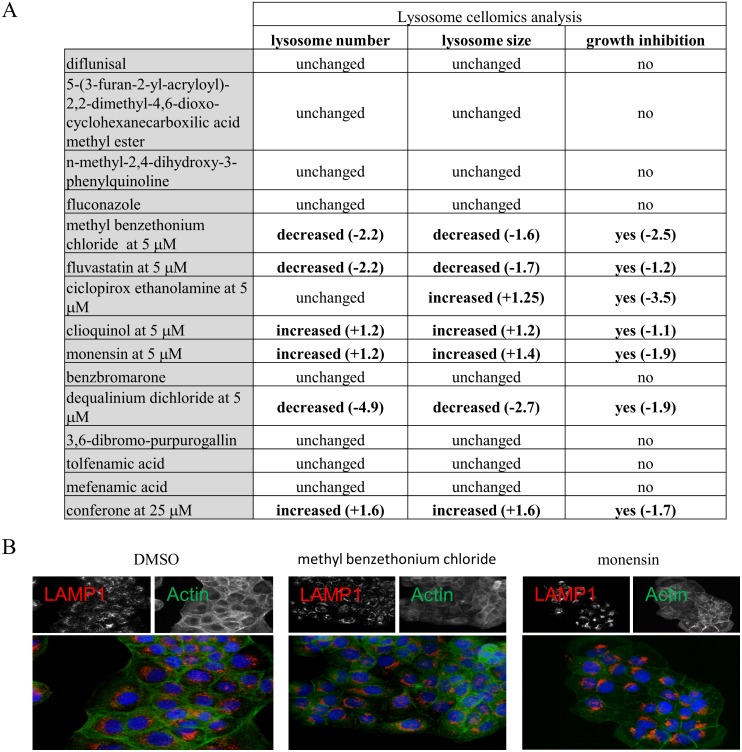
Lysosome cellomics analysis. (A) Bio-imaging cellomics analysis of lysosome number and size, and HaCat cell count. Numbers in brackets indicate the fold variation relative to DMSO treated cells. Only significant decrease or increase are shown, and were obtained from 6 replicates (p-value < 0.0001). Compounds were tested at 5, 25 and 50 μM. The minimal concentration causing abnormal lysosome number and/or size is indicated for each of the active compounds. Fluconazole was used as a control, and the order of the compounds relates to the order in Fig 3. (B) Immunofluorescence was performed using DAPI to detect the cell nucleus and anti-LAMP1 antibody to detect lysosomes. Methyl benzethonium chloride was tested at 5 μM and monensin at 50 μM.

The mammalian lysosome is equivalent to the yeast vacuole, and agents that alter the vacuole could also perturb lysosome structure and/or function. We therefore, examined the effect of the VDAs on lysosomal size and number in mammalian cells using a Cellomics-based imaging assay. Briefly, immortalized human keratinocyte (HaCaT) cells were treated with the 14 VDA agents that caused vacuolar morphological defects, at a 5, 25, and 50 μM for 24 hours. Cells were then stained for lysosome-associated membrane protein 1 (LAMP1), an integral component of the lysosomal membrane, and observed by immunofluorescence. Of the 14 VDA compounds, 7 resulted in abnormal lysosome number or size (Fig 5). Abnormal lysosome phenotypes were most often associated with decreased cell number, suggesting that many of these compounds are cytostatic or cytotoxic to the HaCaT cells. None of these 7 compounds reduced levels of the mature form of the soluble lysosomal protease cathepsin B by immunoblot, suggesting that delivery of cathepsin B and thus trafficking from the Golgi to the lysosome was unaffected (data not shown).

## Discussion

The fungal vacuole is a highly dynamic and multifunctional organelle that plays a central role in a diverse set of cellular responses [49]. However, the mechanisms by which vacuolar function is regulated and coordinated with other aspects of fungal physiology, remain poorly understood. What is clear is that severe disruption of vacuolar integrity is sufficient to render at least two major human fungal pathogens, *C*. *albicans* and *C*. *neoformans* unable to colonize or invade mammalian tissue. Disruption of specific vacuolar membrane trafficking steps can also sensitize *C*. *albicans* to the most widely used class of antifungals, the azoles [50,51]. The goal of this study was to identify chemical probes that disrupt normal vacuolar function in *C*. *albicans*. Such probes can be used as tools to investigate the mechanisms by which vacuolar function is integrated with other aspects of fungal physiology, and potentially provide useful experimental therapeutics.

To date, there have been only limited attempts to identify chemical agents that perturb normal vacuolar function. Two studies have utilized the non-pathogenic yeast *Saccharomyces cerevisiae* to identify compounds that interfere with normal vacuolar acidification [52,53]. Another yeast-based study by Zouhar *et al*. [54] identified fourteen compounds that induce missorting of the vacuolar protease CPY into the culture supernatant. This **v**acuolar **p**rotein **s**orting (*vps*) phenotype is characteristic of impaired membrane trafficking between the Golgi and the vacuole [43,44]. We used the CPP-Nluc missorting assay to determine which of the VDAs identified herein are likely to disrupt Golgi-vacuole trafficking. This assay is based on a similar principle to that used in the Zouhar *et al*. study [54], except missorting of the vacuolar protein into the culture supernatant was detected using luciferase activity rather than antibody based methods. None of the VDAs we identified using the pigmentation screen induced the missorting phenotype of our *vps21Δ/Δ* mutant, suggesting that their primary mechanism of action is unlikely to be disruption of vacuolar trafficking pathways from the Golgi.

The primary screen that we utilized, based on the intra-vacuolar accumulation of the AIR pigment, is simple, requiring no exogenous dyes, antibodies or other manipulation of the cells, and has the potential to identify compounds that act via several distinct mechanisms. Intuitively, we might expect the VDAs identified using our screen to act through one of several mechanisms to reduce pigment accumulation within the *ade2*^*-*^ mutant, including: 1). Blocking vacuolar inheritance or biogenesis; 2). Direct physical interactions with the vacuolar membrane that compromise lipid bilayer integrity; 3). Altering the lipid composition and thus physicochemical properties of the vacuolar membrane; 4). Direct inhibition of the ABC transporters responsible for the bioaccumulation of the AIR pigment within the vacuole; and 5). Disruption of membrane trafficking pathways that deliver these ABC transporters to the vacuolar membrane. Accordingly, the relatively high ‘hit’ rate in our primary screen (1.5%) likely reflects the broad functional basis of the pigmentation assay and the large number of physiological processes that directly or indirectly impinge upon normal vacuolar function and integrity. It probably also reflects the chemical libraries selected that are highly enriched for biologically active molecules.

By far the largest class of ‘hit’ compounds identified was the nine azoles, well characterized antifungal drugs that inhibit the synthesis of ergosterol, a lipid that regulates membrane bilayer fluidity. The statin drug fluvastatin, which blocks sterol biosynthesis through inhibition of HMG-CoA reductase, was also identified as a VDA in our screen, further supporting the importance of fungal sterol content in the maintenance of vacuolar integrity. While the fungal vacuole is typically considered to have only a low concentration of ergosterol relative to the plasma membrane [55,56], it is perhaps not surprising that its depletion would cause loss of vacuolar integrity. We have previously reported that severe vacuolar fragmentation occurs as an early consequence of fluconazole treatment, preceding the inhibition of *C*. *albicans* growth [25]. Similarly, genetic suppression of the azoles target enzyme, Erg11p, also results in massive vacuolar disruption. The importance of ergosterol is further supported by data from the non-pathogenic yeast *S*. *cerevisiae*, where several ergosterol biosynthetic mutants have been noted as having an abnormal vacuole morphology [57,58], and ergosterol has been shown to be required for homotypic vacuole-vacuole fusion in an *in vitro* biochemical assay [58]. It is highly likely that other classes of small molecules that affect the cells fatty acid, phospholipid, or sphingolipid composition will also have significant effects upon vacuolar morphology and function.

Our screen also identified the ionophore monensin, which is known to disrupt the acidification of, and membrane trafficking through post-Golgi compartments within the endosomal network, resulting in abnormal swelling of these organelles [59,60]. Another VDA ‘hit’ worthy of note is benzbromarone, which was previously identified in a screen conducted by Chan *et al*. [52] as causing cytoplasmic acidification in yeast. While benzbromarone did not seem to directly inhibit the vacuolar proton pump (V-ATPase) activity *in vitro*, our data suggest the observed cytoplasmic acidification may relate somehow to abnormal vacuolar function.

Intriguingly a group of three NSAIDs, tolefenamic and mefenamic acids and diflunisal, were also identified as VDAs, and caused a range of vacuolar-related defects, yet none impacted lysosomal integrity. Most NSAIDs primarily act through the inhibition of cyclooxygenase (COX) enzymes of mammals to block the production of inflammatory prostaglandins and thromboxanes. However, several previous studies have reported a variety of NSAIDs to have biological activity upon *C*. *albicans* including the inhibition of yeast-hypha morphogenesis and biofilm formation, as well as inducing azole sensitivity [61,62], all phenotypes that have been associated with endosomal trafficking mutants [24,40,50,51,63]. This includes a study with flufenamic acid, an analog of tolfenamic and mefenamic acids, and which only marginally failed to meet the VDA selection criterion in our primary screen [64]. Thus while the specific mechanisms and molecular targets by which the NSAIDs exert their effect on fungi have remained obscure, it seems likely that at least a sub-set of NSAIDs directly or indirectly interfere with the normal function of the endosomal network. Recent developments have revealed that in addition to inhibition of COX enzymes, many NSAIDs directly associate with membrane phospholipids, altering membrane fluidity and permeability [65,66]. Notably, under the conditions that vacuolar integrity was examined, none of the NSAIDs described here had a profound impact on *C*. *albicans* growth, as would be expected to result from general cellular membrane disorder, suggesting some degree of specificity in targeting the vacuolar membrane. This could be explained by the highly acidified nature of the vacuoles interior, as the membrane association of many NSAIDs is pH dependent [66]. Most NSAIDs are hydrophobic weak acids, properties that facilitate their entry into inflamed tissues where pH is lowered, thus pH is a crucial determinant of their ionization state and in turn partitioning into the hydrophobic interiors of biological membranes. Variations in membrane lipid composition could also favor the interaction of the above three NSAIDs with the vacuolar membrane.

We anticipate that chemical agents that disrupt normal vacuolar function should compromise the pathogenicity of *C*. *albicans* through sensitizing the fungal cells to physiological stress and the inhibition of hyphal growth, a key determinant of *C*. *albicans* pathogenicity. As anticipated, the majority of the VDAs identified in this study adversely affect *C*. *albicans* hyphal growth. However, establishing the antifungal potential of each VDA compound will necessitate a more extensive characterization of their biological activity on an individual basis. This should include determining their precise mechanism of action, effect on fungal physiology, pathogenicity, and potential synergy with established antifungal drugs.

## Supporting information

S1 Fig Pigmentation, CMAC accumulation, and growth in presence of the VDAs. CAI8 cells for pigmentation and CAI4, pLux strain for CMAC accumulation were grown for 24h at 30°C in presence of the indicated drug. Stacked data are shown as percentage of pigmentation (488nm excitation and 569nm emission; white bars), CMAC accumulation (354nm excitation and 469nm emission; checker pattern) and growth (OD600nm; black bars) compare to DMSO-only control. *The full name of the compound is 5-(3-furan-2-yl-acryloyl)-2,2-dimethyl-4,6-dioxo- cyclohexanecarboxilic acid methyl ester.
(PDF)

S2 Fig Nluc-based assay as an indicator of vacuolar trafficking defect. Luminescence measurements were performed from the supernatant of cultures, of cells expressing Nluc or CPP-Nluc, after 24h of treatment at 30°C. All compounds were tested at 5 and 25 μM, and only those that had a significant positive or negative CPP-Nluc over Nluc ratio are represented (three replicates per compound). The strain lacking *VPS21* was used as a positive control for vacuolar trafficking defect and CPP-Nluc missorting.
(PDF)

S3 Fig Hyphal cell growth imaging in presence of the VDAs. *Candida albicans* strain expressing GFP-*YPT72* fusion, which localizes at the vacuolar membrane, was grown in low glucose complete medium at 37°C for 24h in presence of VDAs at 50 μM unless otherwise stated or DMSO (0.5% final). Images were acquired using a Cytation5 imaging reader. The scale bar represents 20 mm.
(ZIP)

S1 Table Primers used in this study. (PDF)

S2 Table Strains used in this study. (PDF)
