# Supplementary figures and images for: Identification of small molecules that disrupt vacuolar function in the pathogen Candida albicans
Source: PLoS One. 2017 Feb 2;12(2):e0171145. doi: 10.1371/journal.pone.0171145 (PMC5289544; doi:10.1371/journal.pone.0171145)

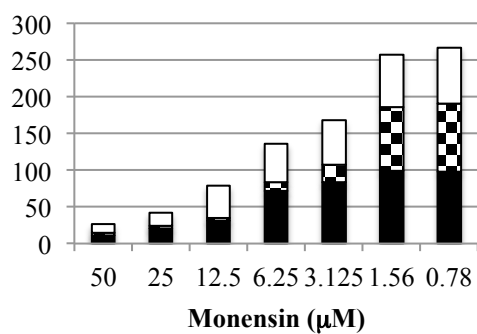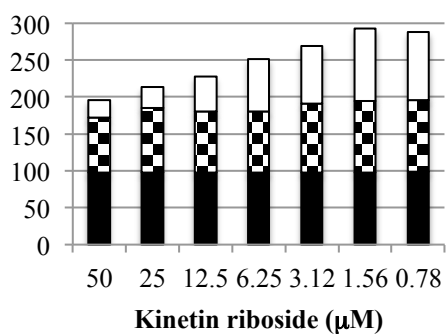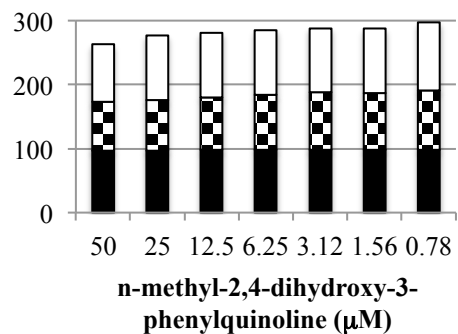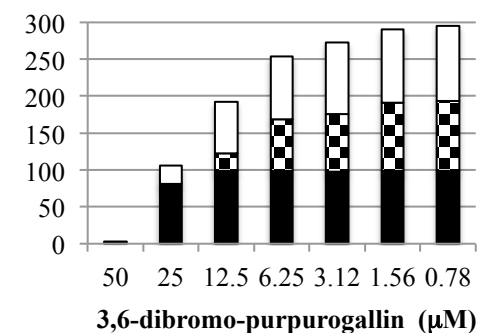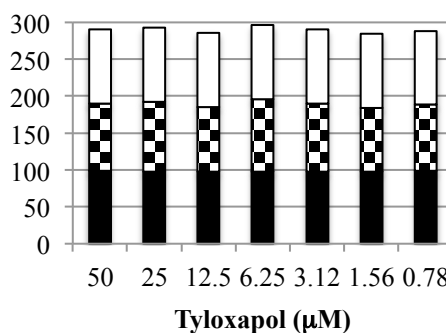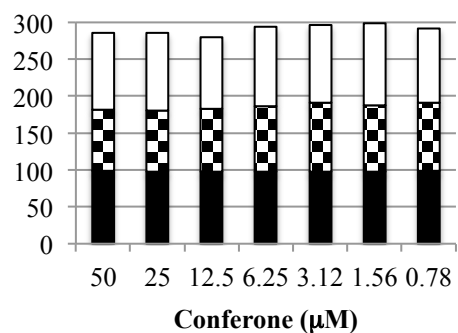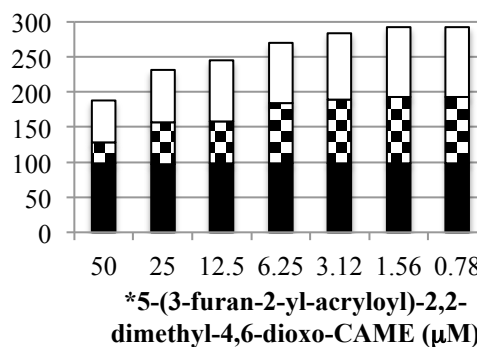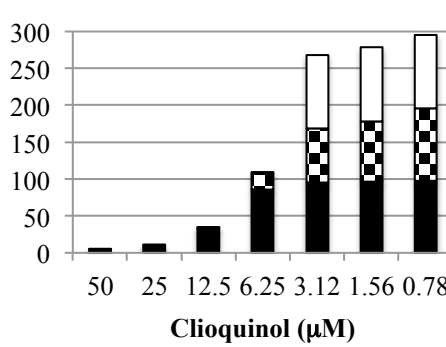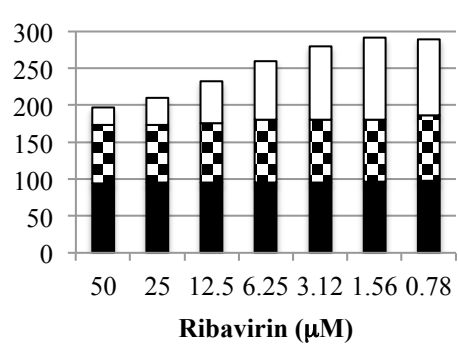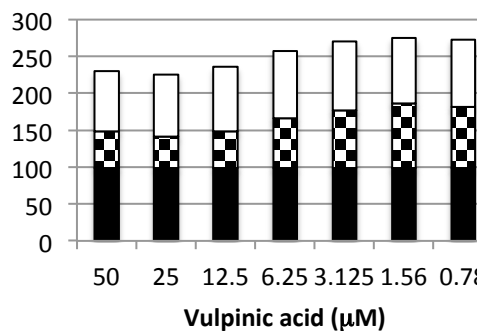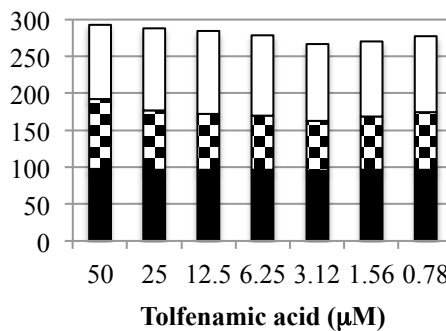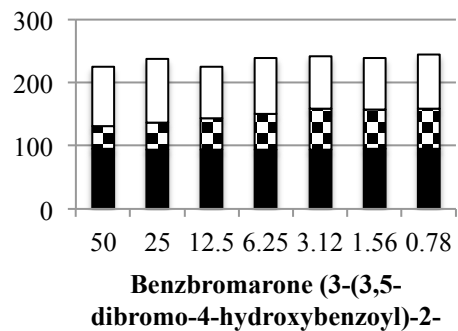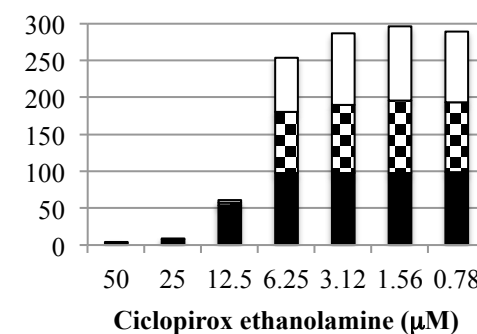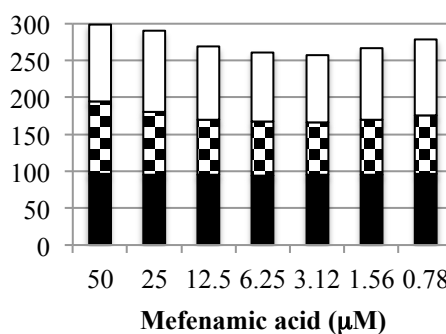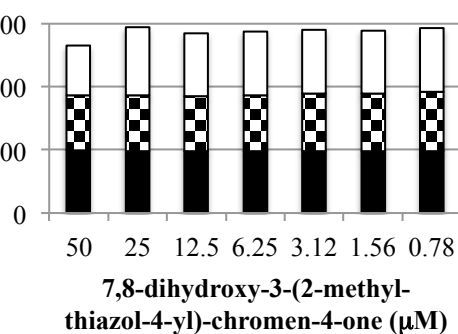

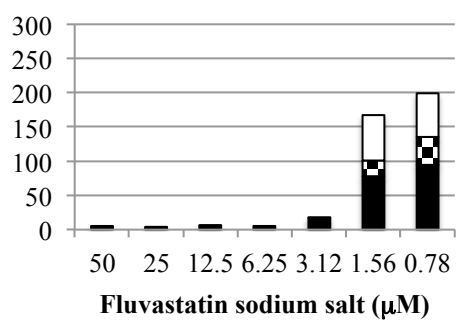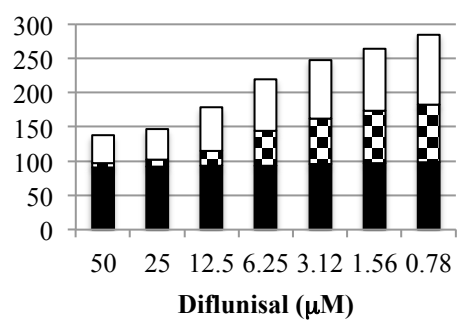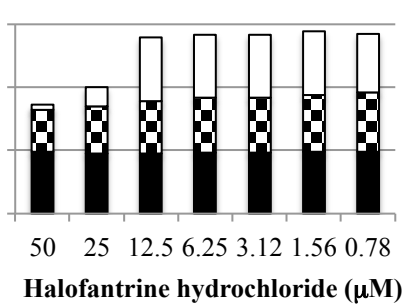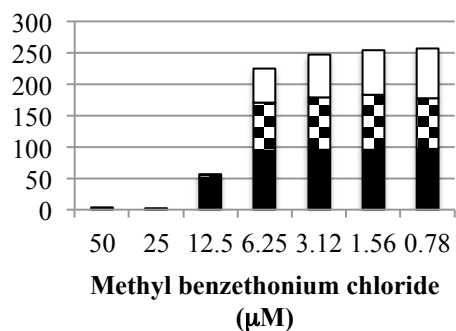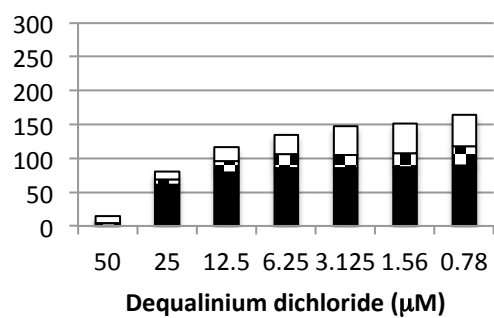

Supplement: S1 Fig — CAI8 cells for pigmentation and CAI4, pLux strain for CMAC accumulation were grown for 24h at 30°C in presence of the indicated drug. Stacked data are shown as percentage of pigmentation (488nm excitation and 569nm emission; white bars), CMAC accumulation (354nm excitation and 469nm emission; checker pattern) and growth (OD600nm; black bars) compare to DMSO-only control. *The full name of the compound is 5-(3-furan-2-yl-acryloyl)-2,2-dimethyl-4,6-dioxo- cyclohexanecarboxilic acid methyl ester. (PDF) [file pone.0171145.s001.pdf]

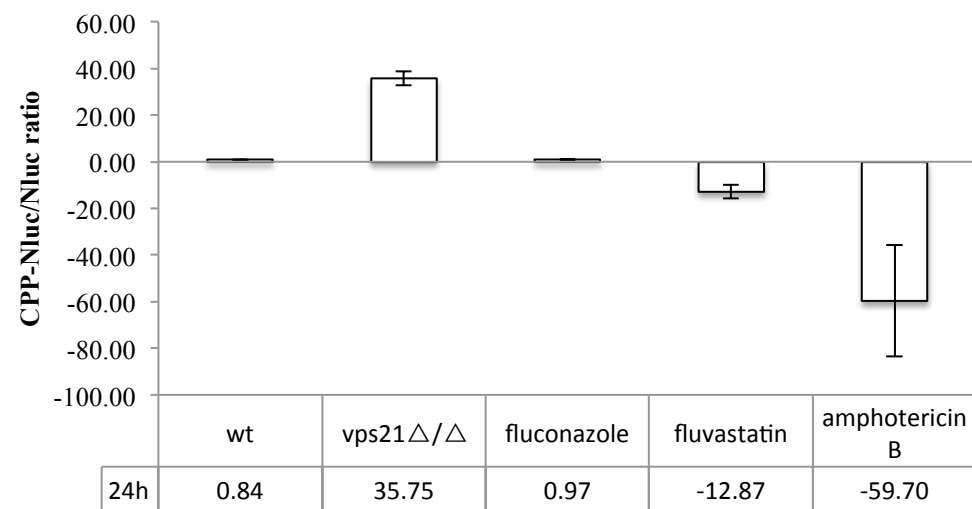

Supplement: S2 Fig — Luminescence measurements were performed from the supernatant of cultures, of cells expressing Nluc or CPP-Nluc, after 24h of treatment at 30°C. All compounds were tested at 5 and 25 μM, and only those that had a significant positive or negative CPP-Nluc over Nluc ratio are represented (three replicates per compound). The strain lacking VPS21 was used as a positive control for vacuolar trafficking defect and CPP-Nluc missorting. (PDF) [file pone.0171145.s002.pdf]
