# Supplementary material for: Identification of small molecules that disrupt vacuolar function in the pathogen Candida albicans
Source: PLoS One. 2017 Feb 2;12(2):e0171145. doi: 10.1371/journal.pone.0171145 (PMC5289544; doi:10.1371/journal.pone.0171145)
Supplement: S3 Fig — Candida albicans strain expressing GFP-YPT72 fusion, which localizes at the vacuolar membrane, was grown in low glucose complete medium at 37°C for 24h in presence of VDAs at 50 μM unless otherwise stated or DMSO (0.5% final). Images were acquired using a Cytation5 imaging reader. The scale bar represents 20 mm. (ZIP) [file pone.0171145.s003.zip › S3 Fig.pdf]

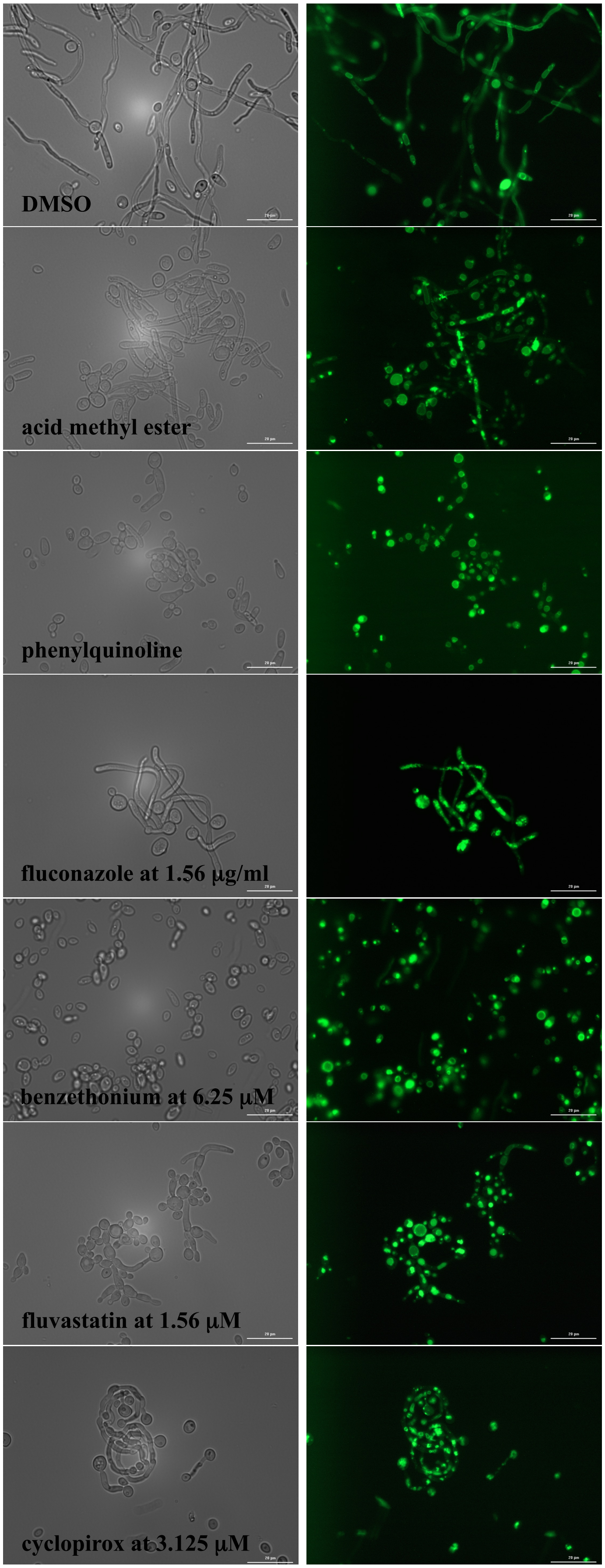

- Long hyphae
- Vacuoles occupying a large proportion of the hyphal cells

- Reduced hyphal growth
- Vacuolar distribution and shape are disturbed

- No hyphae
- Normal distribution of vacuoles

- Most cells have switched to germ tube
- Vacuolar distribution and shape are disturbed

- No hyphae, no germ tubes
- Normal distribution of vacuoles

- No hyphae, some elongated Cells
- Vacuolar distribution and shape are disturbed in some cells

- Elongated phenotype
- Vacuolar distribution and shape are disturbed

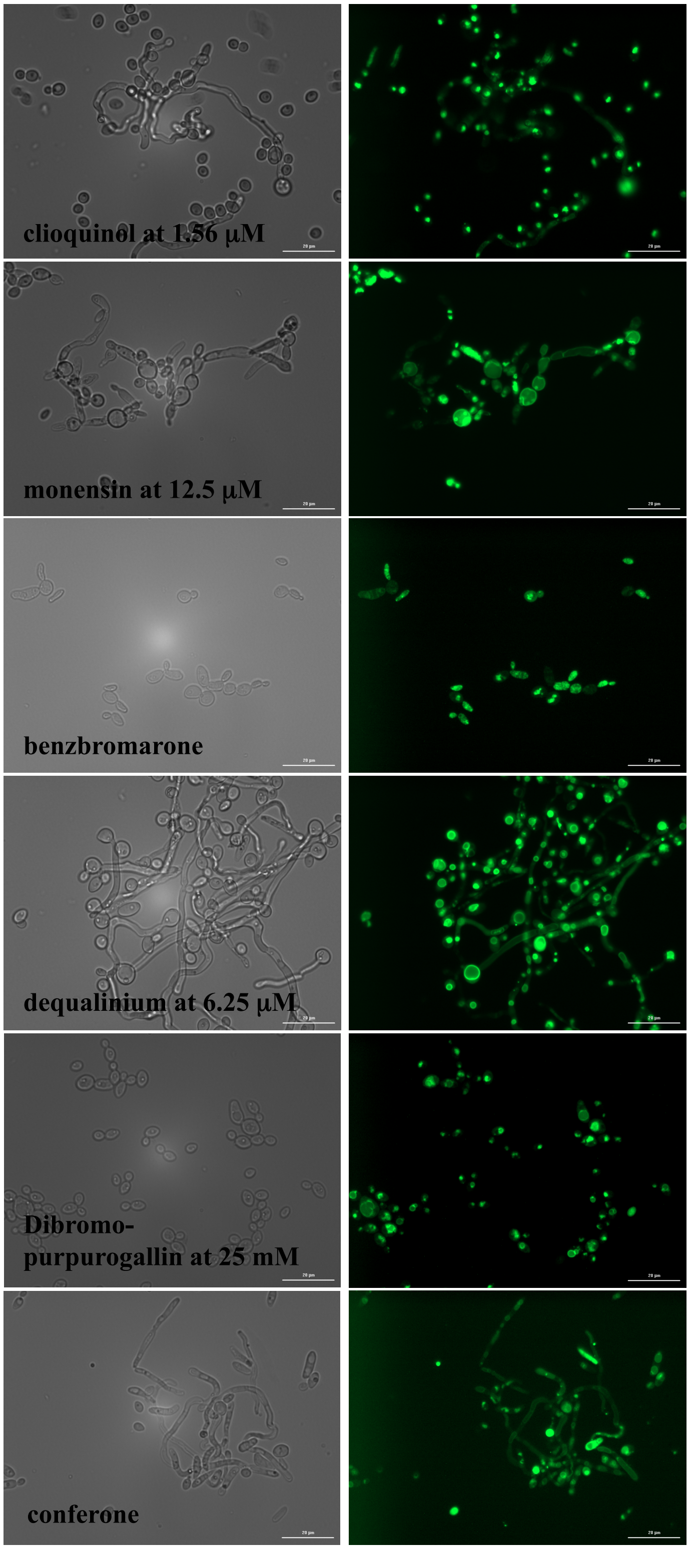

- Some elongated phenotype
- Vacuolar distribution and shape are disturbed

- Some elongated phenotype
- Vacuolar distribution and shape are disturbed

- No hyphae
- Vacuolar distribution and shape are disturbed

- Elongated growth
- Disturbed distribution of vacuoles

- No hyphae
- Vacuolar distribution and shape are disturbed

- Some elongated phenotype
- Normal distribution of the vacuoles
