# Supplementary material for: Identification of small molecules that disrupt vacuolar function in the pathogen Candida albicans
Source: PLoS One. 2017 Feb 2;12(2):e0171145. doi: 10.1371/journal.pone.0171145 (PMC5289544; doi:10.1371/journal.pone.0171145)
Supplement: S1 Table — (PDF) [file pone.0171145.s004.pdf]

Primer	Sequence (5' to 3')
mChORFF-EagI	TCACGGCCGATGGTTTCAAAAGGTGAAGAAG
mChORFR-MluI	TCAACGCGTTTATTTATATAATTCATCCATACCACC
ACT1prF-BamI	TCATCAGGATCCCCAGCCTCGTTTATAATAAACTTAGTC
ADH1-3'UTRR-ApaI	TCATCAGGGCCCATCAATGCCAGAGATCAAACC
YPT72DISF	AACGATACAGAGTTTAATTTAATTTAATTCAATTCAATTTAATTAATTAATCATATACACTTAATTTTCATGTGGAATTGTGAGCGGATA
YPT72DISR	AAACGAATTCGTGATATCTATTTGTTCTCTTTCTTCACCTGTGTATTTGAAATTGATTGTTATATTATTATTTCCCAGTCACGACGTT
YPT72AMPR2	TCATCAGGATCCATTAAAACTGGTAATGATTTGG
YPT72DETF	TGTTGCATTTTATCGAGGGGC
YPT72DETR	CAGGCACTCGCCTCCGAGTCC
VPS11DISF	TTCTCCTTCGAGGACAATATCATCTCCTCTATTATCATTATCATCATGGAGACAGTTCCAATTGTTTGATTGTGGAATTGTGAGCGGATA
VPS11DISR	CTATTGATCTTCCATTACACCTTTACCAATATAATCAGAAACAAATTTAAATTTATCATTACTAGAATGCTTTCCCAGTCACGACGTT
VPS11AMPR2	TCAGGATCCAAATTACTGCATGAGAACACC
VPS11DETF3	GTAGTGGTACAACAGGAATCGG
VPS11DETR3	GATTTATCAATGCCATGATCGG
URA3INTF2	TTATACCATCCAAATCCCGCG
LUXINTDETF	CTGACCTTTAGTCTTTCCTGC

LUXINTDETR	CAGTAGTACTTGTTGTTGTATCG

Engineered restriction sites are underlined.
