# Supplementary material for: Identification of small molecules that disrupt vacuolar function in the pathogen Candida albicans
Source: PLoS One. 2017 Feb 2;12(2):e0171145. doi: 10.1371/journal.pone.0171145 (PMC5289544; doi:10.1371/journal.pone.0171145)
Supplement: S2 Table — (PDF) [file pone.0171145.s005.pdf]

**Strain**	**Parent strain**	**Relevant Genotype**	**Reference**
SC5314		Wild type	(1)
CAI4	SC5314	*ura3Δ:imm434/ura3Δ:imm434 iro1Δ:imm434/iro1Δ:imm434*	(2)
CAI8	CAI4	*ura3Δ:imm434/ura3Δ:imm434 iro1Δ:imm434/iro1Δ:imm434 ade2:hisG/ade2:hisG*	(2)
CAI8 + pLUX	CAI8	*ade2Δ:hisG/ade2::hisG ura3Δ/ura3Δ:URA3-IRO1*	This study
BWP17	CAI4	*ura3Δ:imm434/ura3Δ:imm434 iro1Δ:imm434/iro1Δ:imm434 his1Δ:hisG/his1Δ:hisG arg4Δ:hisG/arg4Δ:hisG*	(3)
C8vps11Δ/Δ	CAI8	*ade2:hisG/ade2:hisG vps11Δ:dpl200/vps11Δ: dpl200 ura3:Δ/ura3Δ:URA3-IRO1*	This study
C8ypt72Δ/Δ	CAI8	*ade2:hisG/ade2:hisG ypt72Δ:dpl200/ypt72Δ: dpl200 ura3Δ/ura3Δ:URA3-IRO1*	This study
YH1	BWP17	*ura3Δ/ura3Δ:URA3-IRO1 his1Δ/Δ arg4Δ/Δ ypt72Δ:ARG4/ypt72Δ:HIS1*	(4)
YR1	BWP17	*ura3Δ/ura3Δ:URA3-IRO1-YPT72 his1Δ/Δ arg4Δ/Δ ypt72Δ:ARG4/ ypt72Δ:HIS1*	(4)
GPH1	BWP17	*ura3Δ/ura3Δ arg4Δ/arg4Δ his1Δ/his1Δ:pGEMHIS1 vps11Δ:ARG4/vps11Δ:URA3*	(5)
GPR1	BWP17	*ura3Δ/ura3Δ arg4Δ/arg4Δ his1Δ/his1Δ:pGEMHIS1-VPS11 vps11Δ:ARG4/vps11Δ:URA3*	(5)
CAI4 + pKE1-NLUC	CAI4	*ura3Δ/ura3Δ:pNLUC-URA3-IRO1*	(6)
CAI4 + pKE1-CPP-NLUC	CAI4	*ura3Δ/ura3ΔpCPP-NLUC-URA3-IRO1*	(6)
CAI4 + pKE1-GFP-*YPT72*	CAI4	*ura3Δ/ura3ΔpGFP-YPT72-URA3-IRO1*	(7)
GP100	BWP17	*ura3Δ/ura3Δ:pKE1-mCh-URA3-IRO1 his1Δ/his1Δ:pKE2-GFP-YPT72-HIS1 arg4Δ/arg4Δ:pRSARG4ΔSpeI*	This study

1. **Gillum AM, Tsay EY, Kirsch DR.** 1984. Isolation of the Candida albicans gene for orotidine-5'-phosphate decarboxylase by complementation of S. cerevisiae ura3 and E. coli pyrF mutations. Mol Gen Genet **198:**179-182.
2. **Fonzi WA, Irwin MY.** 1993. Isogenic strain construction and gene mapping in Candida albicans. Genetics **134:**717-728.
3. **Wilson RB, Davis D, Mitchell AP.** 1999. Rapid hypothesis testing with Candida albicans through gene disruption with short homology regions. J Bacteriol **181:**1868-1874.

4. **Johnston DA, Eberle KE, Sturtevant JE, Palmer GE.** 2009. Role for endosomal and vacuolar GTPases in Candida albicans pathogenesis. Infect Immun **77:**2343-2355.
5. **Palmer GE, Cashmore A, Sturtevant J.** 2003. Candida albicans VPS11 is required for vacuole biogenesis and germ tube formation. Eukaryot Cell **2:**411-421.
6. **Luna-Tapia A, Peters BM, Eberle KE, Kerns ME, Foster TP, Marrero L, Noverr MC, Fidel PL, Jr., Palmer GE.** 2015. ERG2 and ERG24 Are Required for Normal Vacuolar Physiology as Well as Candida albicans Pathogenicity in a Murine Model of Disseminated but Not Vaginal Candidiasis. Eukaryot Cell **14:**1006-1016.
7. **Luna-Tapia A, Kerns ME, Eberle KE, Jursic BS, Palmer GE.** 2015. Trafficking through the late endosome significantly impacts Candida albicans tolerance of the azole antifungals. Antimicrob Agents Chemother **59:**2410-2420.
